# CHOP Chemotherapy for Aggressive Non-Hodgkin Lymphoma with and without HIV in the Antiretroviral Therapy Era in Malawi

**DOI:** 10.1371/journal.pone.0150445

**Published:** 2016-03-02

**Authors:** Satish Gopal, Yuri Fedoriw, Bongani Kaimila, Nathan D. Montgomery, Edwards Kasonkanji, Agnes Moses, Richard Nyasosela, Suzgo Mzumara, Carlos Varela, Maria Chikasema, Victor Makwakwa, Salama Itimu, Tamiwe Tomoka, Steve Kamiza, Bal M. Dhungel, Fred Chimzimu, Coxcilly Kampani, Robert Krysiak, Kristy L. Richards, Thomas C. Shea, N. George Liomba

**Affiliations:** 1 UNC Project-Malawi, Lilongwe, Malawi; 2 Lineberger Comprehensive Cancer Center, Chapel Hill, United States of America; 3 University of North Carolina Department of Pathology and Laboratory Medicine, Chapel Hill, United States of America; 4 University of Malawi College of Medicine, Blantyre, Malawi; 5 Kamuzu Central Hospital, Lilongwe, Malawi; 6 Weill Cornell Medical College, Ithaca, United States of America; Mayo Clinic, UNITED STATES

## Abstract

There are no prospective studies of aggressive non-Hodgkin lymphoma (NHL) treated with CHOP in sub-Saharan Africa. We enrolled adults with aggressive NHL in Malawi between June 2013 and May 2015. Chemotherapy and supportive care were standardized, and HIV+ patients received antiretroviral therapy (ART). Thirty-seven of 58 patients (64%) were HIV+. Median age was 47 years (IQR 39–56), and 35 (60%) were male. Thirty-five patients (60%) had stage III/IV, 43 (74%) B symptoms, and 28 (48%) performance status ≥2. B-cell NHL predominated among HIV+ patients, and all T-cell NHL occurred among HIV- individuals. Thirty-one HIV+ patients (84%) were on ART for a median 9.9 months (IQR 1.1–31.7) before NHL diagnosis, median CD4 was 121 cells/μL (IQR 61–244), and 43% had suppressed HIV RNA. HIV+ patients received a similar number of CHOP cycles compared to HIV- patients, but more frequently developed grade 3/4 neutropenia (84% vs 31%, p = 0.001), resulting in modestly lower cyclophosphamide and doxorubicin doses with longer intervals between cycles. Twelve-month overall survival (OS) was 45% (95% CI 31–57%). T-cell NHL (HR 3.90, p = 0.017), hemoglobin (HR 0.82 per g/dL, p = 0.017), albumin (HR 0.57 per g/dL, p = 0.019), and IPI (HR 2.02 per unit, p<0.001) were associated with mortality. HIV was not associated with mortality, and findings were similar among patients with diffuse large B-cell lymphoma. Twenty-three deaths were from NHL (12 HIV+, 11 HIV-), and 12 from CHOP (9 HIV+, 3 HIV-). CHOP can be safe, effective, and feasible for aggressive NHL in Malawi with and without HIV.

## Introduction

Non-Hodgkin lymphoma (NHL) is increasing in sub-Saharan Africa (SSA) [1–3], but clinical features and outcomes are not well described. Reports have typically been retrospective, with many excluded or untreated patients, and high loss to follow-up. Descriptions have also often been before widespread availability of antiretroviral therapy (ART) for HIV.

NHL diagnosis in SSA is frequently based on fine needle aspirates (FNAs) without immunohistochemistry (IHC), flow cytometry, or molecular tools [4–6]. NHL is therefore often treated as a single entity with CHOP being universally applied, given pathology limitations, restricted chemotherapy formularies, and settings where more intense or complex regimens are impractical. Even in resource-rich countries, CHOP remains a standard backbone for many NHL subtypes. The most important advance has been adding rituximab for CD20-positive NHL, which is neither widely available nor well-studied in SSA.

Despite widespread use, there is no prospective description of first-line CHOP for adults with NHL in SSA. In the only clinical trial for this population, CHOP salvage was administered to 11 of 49 HIV-infected NHL patients who failed dose-modified oral chemotherapy in Kenya and Uganda, during 2001–2005 when ART was not widely available [7]. In the modern era, HIV-associated lymphoma patients in resource-rich settings can receive identical treatment with equivalent outcomes to patients without HIV [8–13]. Retrospective descriptions from Uganda and South Africa have also suggested CHOP can be effective, including for HIV-infected individuals receiving ART [14, 15]. Given high infectious burden and limited cancer infrastructure [16], a detailed prospective characterization of CHOP in SSA is important, and was undertaken at a national teaching hospital in Malawi.

## Methods

### Patients and procedures

Kamuzu Central Hospital (KCH) is a cancer referral center for eight million people in Malawi, a country which has 10% HIV prevalence, 67% ART coverage, and an annual gross domestic product per capita of 314 US dollars [17, 18]. The KCH Lymphoma Study is an ongoing prospective cohort approved by the University of North Carolina Institutional Review Board and Malawi National Health Sciences Research Committee. Through active case finding across all hospital departments and referring clinics, adults and children with newly diagnosed lymphoma were invited to participate after written informed consent. Informed consent forms and consent procedures were approved by ethics committees in the US and Malawi. All diagnoses were pathologically confirmed using tissue biopsies whenever possible, supported by IHC and weekly telepathology consultation involving 2–4 US and Malawian pathologists [19]. Available IHC stains included CD3, CD20, CD30, CD45, CD138, Ki-67, terminal deoxynucleotidyl transferase (TDT), and latency-associated nuclear antigen (LANA). Other stains included synaptophysin and AE1/AE3 to distinguish lymphomas from neuroendocrine or epithelial tumors when morphology is equivocal. Definitively distinguishing Burkitt lymphoma (BL), diffuse large B-cell lymphoma (DLBCL), and overlapping subtypes was difficult without cytogenetic and molecular tools. These subtypes were therefore grouped as aggressive B-cell NHL and treated with CHOP, since infusional or more intensive regimens for BL or other NHL subtypes were not feasible. All diagnostic specimens were shipped to the US for final classification, although results were not available in time to guide frontline therapy.

Patients underwent comprehensive baseline evaluation including chest x-ray, abdominal ultrasound, and bone marrow examination. Cerebrospinal fluid (CSF) cytology was recommended for patients at high risk for leptomeningeal involvement (>1 extranodal site and elevated lactate dehydrogenase (LDH); involvement of bone marrow, testicular, epidural, ocular, breast, or paranasal sinus sites; or Ki67 >90% suggesting BL or similarly aggressive B-cell NHL). Patients received longitudinal follow-up with active tracing and transportation reimbursement to promote retention. Response was assessed using standardized criteria incorporating physical exam, chest x-ray, and abdominal ultrasound. For this report, we focused on adults >18 years with newly diagnosed aggressive NHL enrolled between June 1, 2013 and May 31, 2015.

There are no formal cancer treatment guidelines in Malawi. However, efforts to standardize care have been ongoing. CHOP was administered every 21 days with cyclophoshamide 750 mg/m^2^ day 1, doxorubicin 50 mg/m^2^ day 1, vincristine 1.4 mg/m^2^ (maximum 2 mg) day 1, and prednisone 60 mg/m^2^ (maximum 100 mg) days 1–5. Prior to each cycle, an absolute neutrophil count (ANC) of 1.0x10^3^/μL and platelet count of 100x10^3^/μL were recommended. Intrathecal methotrexate 12.5 mg and hydrocortisone 50 mg were recommended for patients at high risk of leptomeningeal involvement as above. There is no radiotherapy in Malawi, and six cycles were administered even for limited-stage disease. Patients who achieved partial response after six cycles, and tolerated treatment without severe adverse events, could receive up to eight cycles.

Blood counts and tests of kidney and liver function were performed on day 1 of each cycle or interim visits as indicated. If blood counts prohibited chemotherapy, they were repeated one week later and treatment proceeded if blood counts allowed. For a first episode of febrile neutropenia or delayed blood count recovery, cyclophosphamide and doxorubicin were reduced by 25%, and for a second episode doses were reduced by 50%, with subsequent dose escalation at the treating physician’s discretion. Hematopoietic growth factors were not available. Patients with age >70 years, performance status >2, and/or baseline cytopenias could receive prephase prednisone and/or ‘mini-CHOP’ with 50% dose reductions of all cytotoxic agents for cycle 1, and subsequent dose escalation at the treating physician’s discretion. Ciprofloxacin prophylaxis was provided during days 8–15 of each cycle for patients with HIV, or patients without HIV >60 years. Cotrimoxazole prophylaxis was continuously provided to all HIV-infected patients. Fluconazole prophylaxis was provided to HIV-infected patients throughout chemotherapy until resolution of neutropenia. Promethazine was provided for antiemesis. Consistent with World Health Organization guidelines and anti-infective prophylaxis provided in US AIDS Malignancy Consortium trials, we continued cotrimoxazole even in patients who developed neutropenia due to demonstrated survival benefits for HIV-infected populations without cancer in SSA through its preventive effects against malaria, bacterial infection, and *Pneumocystis*.All medicines were freely provided by the Ministry of Health, although drug shortages occurred. External funding enabled a back-up supply to mitigate treatment interruptions. Interval illnesses and complications were recorded prior to each cycle, and patients were encouraged to return promptly for interim concerns. Toxicities were graded using National Cancer Institute Common Terminology Criteria for Adverse Events (CTCAE) version 4.0. All HIV-infected patients received ART concurrently with chemotherapy, typically with tenofovir-lamivudine-efavirenz. Patients on ART with virologic failure were referred for adherence counseling and consideration of second-line ART. We reduced vincristine by 50% in patients receiving second-line ritonavir-based ART due to cytochrome P450 3A4 interactions.

### Statistical analysis

Differences between HIV-infected and HIV–uninfected patients were assessed using Fisher’s exact tests, one-way analysis of variance (ANOVA), and Kruskal-Wallis tests. Summative curves for blood counts were derived by plotting median values over time across all patients within each group. Follow-up time was calculated from enrollment or CHOP initiation as specified, until death, loss to follow-up, or administrative censoring on August 31, 2015. Kaplan-Meier curves were used to estimate overall survival (OS), and the log-rank test was used to assess differences in survival between subgroups. Cox proportional hazards were used to estimate unadjusted and adjusted hazard ratios for mortality. All analyses were conducted using Stata version 12.1 (College Station, Texas, USA). A two-sided alpha value of 0.05 was considered statistically significant.

## Results

Sixty-two patients ≥18 years with newly diagnosed aggressive NHL presented to KCH between June 1, 2013 and May 31, 2015, and 59 (95%) patients consented to participate. Of those who presented for care, one patient could not give adequate informed consent, one patient elected to receive treatment in Blantyre, and one patient declined study participation. Four additional patients with aggressive NHL were identified through the pathology laboratory, but could not be contacted despite repeated attempts to engage them in care. One additional enrolled patient had follicular lymphoma with DLBCL transformation, leaving 58 enrolled adults with newly diagnosed aggressive NHL without prior indolent lymphoma.

Baseline characteristics are shown in Table 1. Thirty-seven patients (64%) were HIV-infected. Median age was 47 years [interquartile range (IQR) 39–56] and 35 (60%) were male. Thirty-five patients (60%) had stage III/IV disease, B symptoms were present in 43 (74%), and 28 (48%) had performance status ≥2. Aggressive B-cell NHL predominated among HIV-infected patients, and all aggressive T-cell NHL occurred among HIV-negative individuals. There was a tendency for HIV-negative patients to be older with bulkier disease and higher hemoglobin than HIV-positive patients. HIV-negative patients also more frequently reported symptom durations ≥6 months. Thirty-one (84%) HIV-infected patients were on ART at lymphoma diagnosis for a median 9.9 months (IQR 1.1–31.7). Median CD4 at lymphoma diagnosis was 121 cells/μL (IQR 61–244), median HIV RNA was 3.5 log_10_copies/mL (IQR 1.3–4.8), and 43% had suppressed HIV RNA <400 copies/mL.

**Table 1:** Baseline characteristics for adults with aggressive non-Hodgkin lymphoma in Lilongwe, Malawi, June 2013 to May 2015.

	HIV-positive (n = 37)	HIV-negative (n = 21)	P
Age, years, median (IQR)	47.0 (38.1–50.2)	54.6 (43.9–61.8)	***0*.*044***
Male, n (%)	22 (59.5%)	13 (61.9%)	1.00
Body mass index, kg/m^2^, median (IQR)	20.8 (18.9–24.3)	20.3 (17.7–22.1)	0.39
Histologic diagnosis, n (%)	34 (91.9%)	19 (90.5%)	1.00
Malawi diagnosis^a^			***0*.*014***
Aggressive B-cell lymphoma	37	17	
Aggressive T-cell lymphoma	―	4	
United States diagnosis^b^			0.19
Diffuse large B-cell lymphoma	26	13	
Burkitt lymphoma	2	1	
Plasmablastic lymphoma	2	1	
High-grade B-cell lymphoma NOS	2	1	
Extranodal NK/T-cell lymphoma	―	3	
ALK-negative anaplastic large-cell lymphoma	―	1	
Symptom duration ≥6 months, n (%)	3/36 (8.3%)	7/20 (35.0%)	***0*.*025***
B symptoms, n (%)	26 (70.3%)	17 (81.0%)	0.54
Largest lymph node mass, cm, median (IQR)	10 (6–12)	11 (9–17)	0.056
Performance status ≥2, n (%)	16 (43.2%)	12 (57.1%)	0.41
Stage III/IV, n (%)	24 (64.9%)	11 (52.4%)	0.41
International prognostic index ≥3, n (%)	12 (32.4%)	8 (38.1%)	0.78
White blood cells, 10^3^/μL, median (IQR)	5.7 (4.2–7.6)	5.6 (4.4–8.9)	0.59
Absolute neutrophil count, 10^3^/μL, median (IQR)	2.6 (1.8–4.1)	2.9 (2.5–5.7)	0.14
Hemoglobin, g/dL, median (IQR)	11.0 (9.2–12.4)	12.4 (11.0–13.3)	***0*.*038***
Platelets, 10^3^/μL, median (IQR)	263 (184–402)	290 (232–425)	0.40
Albumin, g/dL, median (IQR)	3.4 (2.9–4.0)	3.2 (3.0–3.8)	0.73
Lactate dehydrogenase, IU/L, median (IQR)^c^	602 (338–1,294)	441 (283–571)	0.11
Abnormal lactate dehydrogenase, n (%)	33 (89.2%)	17 (81.0%)	0.44
Bone marrow involvement, n (%)	5/27 (18.5%)	0/17 (0.0%)	0.14
≥2 extranodal sites, n (%)	6 (16.2%)	4 (19.0%)	1.00
Hepatitis B surface antigen positive, n (%)	6/36 (16.7%)	1/19 (5.3%)	0.40
ART at enrollment, n (%)	31 (83.8%)	―	―
ART duration at enrollment, months, median (IQR)	9.9 (1.1–31.7)		
CD4 count, cells/μL, median (IQR)	121 (61–244)	―	―
HIV RNA, log_10_copies/mL, median (IQR)	3.5 (1.3–4.8)	―	―
HIV RNA <400 copies/mL, n (%)	16 (43.2%)	―	―

IQR = interquartile range. NOS = not otherwise specified. ART = antiretroviral therapy.

^a^Malawi diagnoses reflect limited immunohistochemistry staining and weekly consensus telepathology discussion by United States and Malawi pathologists.

^b^United States diagnoses reflect delayed review of 52 cases with additional immunohistochemistry, fluorescence in situ hybridization, and molecular studies in Chapel Hill.

^c^Laboratory upper limit of normal is 250 IU/L.

Fifty patients (86%) initiated cytotoxic treatment, including 31/37 (84%) HIV-positive and 19/21 (90%) HIV-negative individuals. Median time between enrollment and cytotoxic treatment initiation was 7 days (IQR 4–13). All eight patients (6 HIV+, 2 HIV-) not treated with CHOP died before cytotoxic treatment could be initiated. Eleven of 50 patients (22%) treated with CHOP began treatment with either prephase prednisone or dose-reduced mini-CHOP, including 6/31 (19%) HIV-positive and 5/19 (26%) HIV-negative patients. Four (3 HIV+, 1 HIV-) received prephase prednisone followed by dose-reduced cytotoxic treatment with their first cycle, three HIV-positive patients received prephase prednisone followed by full-dose CHOP, and four HIV-negative patients initiated dose-reduced cytotoxic treatment without prephase prednisone. Of eight patients who initiated dose-reduced cytotoxic treatment, four subsequently proceeded to full-dose chemotherapy, with reasons for initial dose reduction being impaired performance status (5), baseline cytopenias (2), and advanced age (1).

**Fig 1:**
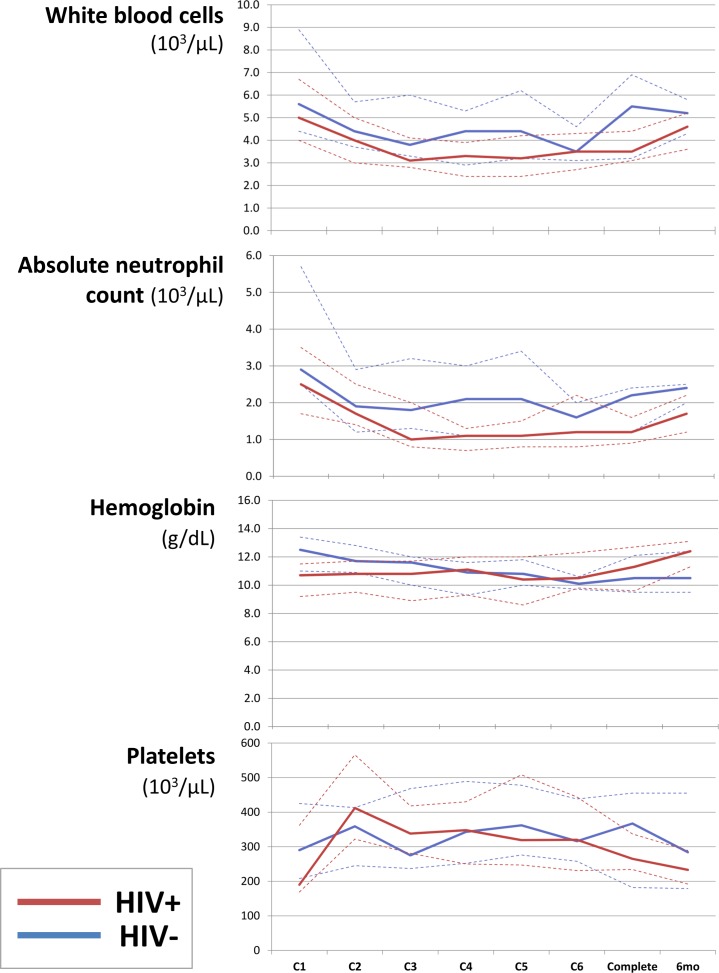
Neutropenia during CHOP chemotherapy in Lilongwe, Malawi.

**Fig 2:**
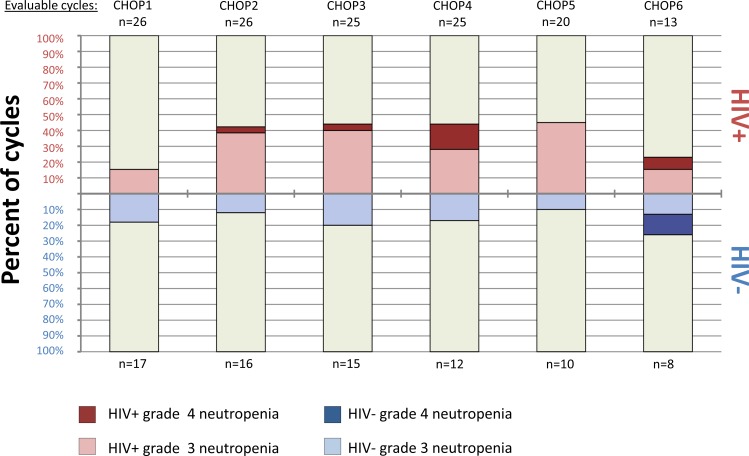
Peripheral blood counts with interquartile ranges during CHOP chemotherapy in Lilongwe, Malawi.

Achieved chemotherapy intensity and toxicities are shown in Table 2. HIV-infected patients received a similar number of CHOP cycles compared to HIV-uninfected patients (median 6 vs 5, p = 0.59), but modestly lower cyclophosphamide and doxorubicin doses per cycle, with a longer interval between cycles. Grade 3/4 neutropenia occurred in more HIV-infected than HIV-uninfected patients (84% vs 31%, p = 0.001). Grade 3/4 anemia occurred in 24% of patients overall without significant differences between HIV-positive and HIV-negative individuals, and only one grade 3/4 thrombocytopenia event occurred in the study population. Of 62 grade 3/4 neutropenia events, 54 (87%) were grade 3 and eight (13%) were grade 4 (Fig 1). For both HIV-infected and HIV-uninfected patients, median ANC remained greater than 1x10^3^/μL throughout treatment (Fig 2). Among those with HIV, median CD4 count increased to 146 cells/μL (IQR 98–265) and median HIV RNA decreased to 1.3 log_10_copies/mL (IQR 1.3–1.6) at six months with 11 of 13 patients (85%) having suppressed HIV RNA <400 copies/mL.

**Table 2:** Treatment course and toxicities during CHOP chemotherapy in Lilongwe, Malawi.

	HIV-positive (n = 31)	HIV-negative (n = 19)	P
Total chemotherapy cycles	150	88	―
Cycles per patient, median (IQR)^a^	6 (4–6)	5 (3–6)	0.59
Days between cycles, median (IQR)	21 (21–27)	21 (20–22)	***0*.*0053***
Cycles delayed ≥7 days, n (%)^b^	32/119 (26.9%)	8/69 (11.6%)	***0*.*016***
Cyclophosphamide dose per cycle, mg/m^2^, median (IQR)^c^	693.0 (555.6–734.8)	721.4 (576.9–750.0)	***0*.*015***
Doxorubicin dose per cycle, mg/m^2^, median (IQR)^d^	48.0 (37.6–49.9)	49.4 (39.3–50.0)	***0*.*042***
Received <6 cycles, n (%)^a^	14/30 (46.7%)	11/19 (57.9%)	0.56
Death	7	6	
Progression	1	2	
Toxicity	5	1	
Social	1	2	
Grade 3/4 neutropenia^e^			
Cycles, n (%)	49/140 (35.0%)	13/83 (15.7%)	***0*.*002***
Patients, n (%)	21/25 (84.0%)	5/16 (31.2%)	***0*.*001***
Grade 3/4 anemia^e^			
Cycles, n (%)	13/140 (8.9%)	6/83 (7.2%)	0.80
Patients, n (%)	7/25 (28.0%)	3/16 (18.8%)	0.71
Grade 3/4 non-hematologic toxicity^e^			
Cycles, n (%)	6/140 (4.3%)	12/83 (14.5%)	***0*.*010***
Patients, n (%)	6/25 (24.0%)	8/17 (47.1%)	0.18

IQR = interquartile range.

^a^Includes 49 patients (30 HIV+, 19 HIV-) who completed first-line CHOP or died as of August 31, 2015.

^b^Excudes first treatment cycles.

^c^Excludes 9 missed cyclophosphamide doses due to stock-out (6 HIV-, 3 HIV+).

^d^Excludes 6 missed doxorubicin doses due to stock-out (4 HIV-, 2 HIV+).

^e^Toxicity assessment includes patients and cycles with subsequent follow-up visits making them evaluable for interim toxicity; deaths occurring out of hospital are separately adjudicated (S1 Table) without evaluation for non-fatal interim toxicity.

Eighteen grade 3/4 non-hematologic toxicities occurred, including nine grade 3 diarrhea/dehydration, two grade 3 abdominal pain, two grade 3 skin infection, one grade 4 cerebral edema, one grade 4 renal failure, one grade 3 small bowel obstruction, one grade 3 hepatitis, and one grade 3 sepsis without neutropenia. Cerebral edema occurred in a patient with systemic and central nervous system NHL progression. Obstructive renal failure, abdominal pain, and small bowel obstruction occurred in patients with abdominal NHL progressing on CHOP. Both grade 3 skin infections occurred when large masses ulcerated during CHOP and became superinfected. Of 38 CHOP cycles delayed at least seven days (31 HIV+, 7 HIV-), 31 were due to delayed ANC recovery, three for personal or transportation issues, three for clinic scheduling, and one for pharmacy stock-out.

**Fig 3:**
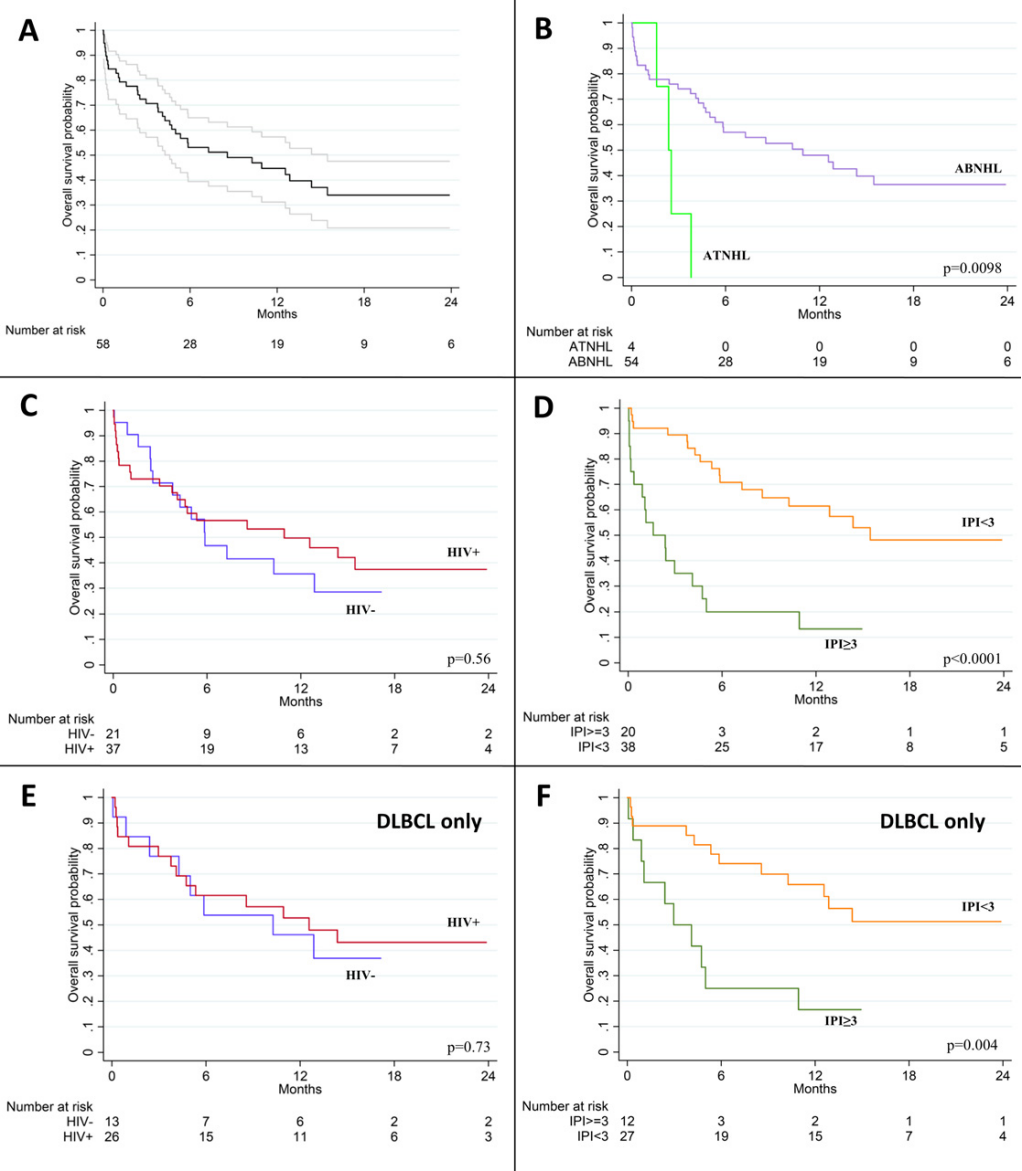
Overall survival for aggressive non-Hodgkin lymphoma in Lilongwe, Malawi. (A) Overall cohort with 95% confidence intervals. (B) Aggressive B-cell non-Hodgkin lymphoma (ABNHL) versus aggressive T-cell non-Hodgkin lymphoma (ATNHL). (C) Stratified by HIV status. (D) Stratified by international prognostic index (IPI). (E) Diffuse large B-cell lymphoma (DLBCL) stratified by HIV status. (F) DBLCL stratified by IPI.

As of August 31, 2015, vital status was known for all 58 patients with no loss to follow-up after a median 14.9 months among patients still alive (IQR 7.8–24.4). Among 49 patients who initiated first-line CHOP and completed treatment or died before the censoring date, 22 (45%) achieved a complete response (CR) after a median 2 CHOP cycles (IQR 1–3) including 15 of 30 (50%) HIV-infected patients and 7 of 19 (37%) HIV-uninfected patients (p = 0.40). One HIV-infected patient was still receiving first-line CHOP and had achieved CR after four cycles. Kaplan-Meier OS curves are shown in Fig 3, and OS estimates are shown in Table 3 for all enrolled patients and patients who initiated cytotoxic treatment. Kaplan-Meier 12-month OS was 45% [95% confidence interval (CI) 31–57%] for the cohort overall. OS was similar for HIV-infected and HIV-uninfected patients, better for those with international prognostic index (IPI) <3, and better for those with aggressive B-cell NHL than T-cell NHL. Among 50 patients who initiated cytotoxic treatment, estimated 12-month OS was 52% (95% CI 37–65%). For cases confirmed as DLBCL in the US, 12-month OS was 51% (95% CI 34–65%) for all patients (n = 39) and 56% (95% CI 38–71%) for those who initiated cytotoxic treatment (n = 35), with similar survival by HIV status and better survival for those with IPI <3.

**Table 3:** Overall survival estimates for aggressive non-Hodgkin lymphoma in Lilongwe, Malawi.

	N	12-month OS (%)	95% CI	24-month OS (%)	95% CI
*Enrolled*^a^					
All	58	44.7	31.2–57.3	34.0	20.9–47.6
HIV+	37	49.7	32.4–64.9	37.4	20.5–54.3
HIV-	21	35.6	15.8–56.1	28.5	10.4–49.9
IPI<3	38	61.5	43.6–75.2	45.1	26.8–61.9
IPI> = 3	20	13.3	2.6–32.7	13.3	2.7–32.7
DLBCL	39	50.6	33.9–65.0	40.8	24.8–56.3
HIV+	26	52.8	31.9–69.9	43.2	23.2–61.7
HIV-	13	46.2	19.2–69.6	36.9	12.5–62.0
IPI<3	27	65.8	44.5–80.6	51.3	29.9–69.2
IPI> = 3	12	16.7	2.6–41.3	16.7	2.6–41.3
*Treated*^b^					
All	50	51.9	36.8–65.1	39.3	24.1–54.1
HIV+	31	59.4	39.3–74.7	44.3	24.2–62.7
HIV-	19	39.4	17.5–60.7	31.5	11.4–54.1
IPI<3	35	66.8	47.8–80.1	48.9	28.7–66.0
IPI> = 3	15	17.8	3.4–41.4	17.8	3.4–41.4
DLBCL	35	56.3	38.2–71.0	45.2	27.4–61.4
HIV+	23	59.6	36.5–76.7	48.2	25.6–67.6
HIV-	12	50.0	20.8–73.6	40.0	13.5–65.7
IPI<3	24	74.1	51.0–87.5	57.1	32.9–75.4
IPI> = 3	11	18.2	2.8–44.2	18.2	2.8–44.2

OS = overall survival. CI = confidence interval. IPI = International Prognostic Index.

^a^Survival measured from enrollment.

^b^Survival measured from cytotoxic treatment initiation.

Risk factors for mortality are shown in Table 4. In unadjusted analyses, T-cell NHL (HR 3.90, p = 0.017), hemoglobin (HR 0.82 per g/dL increase, p = 0.017), albumin (HR 0.57 per g/dL increase, p = 0.019), and IPI (HR 2.02 per unit increase, p<0.001) were associated with mortality. When individual IPI elements were examined, performance status (HR 1.91 per unit increase, p<0.001), LDH (HR 1.04 per 100 IU/L increase, p = 0.002), number of extranodal sites (HR 1.66 per site, p = 0.006), and stage III/IV (HR 2.33, p = 0.025) were associated with mortality. In adjusted analyses, only IPI was associated with mortality (HR 1.77 per unit increase, p = 0.005). HIV was not associated with mortality, and findings were similar among patients confirmed as DLBCL in the US.

**Table 4:** Risk factors for mortality among aggressive non-Hodgkin lymphoma patients in Lilongwe, Malawi.

	Unadjusted hazard ratio	95% confidence interval	P	Adjusted hazard ratio^a^	95% confidence interval	P
*All (n = 58)*						
HIV infection	0.82	0.41–1.61	0.56	0.90	0.39–2.07	0.81
T-cell non-Hodgkin lymphoma	3.90	1.28–11.91	***0*.*017***	3.06	0.74–12.72	0.12
Female sex	0.80	0.40–1.61	0.53	0.57	0.26–1.24	0.16
Hemoglobin, per g/dL	0.82	0.70–0.97	***0*.*017***	0.92	0.74–1.14	0.44
Albumin, per g/dL	0.57	0.36–0.91	***0*.*019***	1.07	0.53–2.17	0.85
Body mass index, per kg/m^2^	0.95	0.86–1.05	0.29	1.00	0.90–1.11	0.98
International prognostic index, per unit	2.02	1.45–2.82	***<0*.*001***	1.77	1.18–2.64	***0*.*005***
Age, per year	1.00	0.97–1.02	0.85	―	―	―
Age >60 years	0.75	0.33–1.73	0.50	―	―	―
Performance status, per unit	1.91	1.45–2.52	***<0*.*001***	―	―	―
Performance status ≥2	4.96	2.34–10.50	***<0*.*001***	―	―	―
Stage III/IV	2.32	1.11–4.85	***0*.*025***	―	―	―
Lactate dehydrogenase, per 100 IU/L	1.04	1.01–1.07	***0*.*002***	―	―	―
Abnormal lactate dehydrogenase	1.81	0.63–5.16	0.27	―	―	―
Extranodal sites, per site	1.66	1.16–2.38	***0*.*006***	―	―	―
≥2 extranodal sites	2.59	1.13–5.96	***0*.*025***	―	―	―
*DLBCL only (n = 39)*						
HIV infection	0.86	0.36–2.05	0.73	1.37	0.50–3.76	0.54
Female sex	1.05	0.44–2.51	0.91	1.99	0.67–5.91	0.21
Hemoglobin, per g/dL	0.89	0.71–1.12	0.32	1.07	0.82–1.41	0.60
Albumin, per g/dL	0.64	0.33–1.23	0.18	0.89	0.35–2.26	0.80
Body mass index, per kg/m^2^	0.97	0.87–1.09	0.65	1.09	0.95–1.25	0.17
International prognostic index, per unit	2.37	1.49–3.77	***<0*.*001***	2.94	1.49–5.83	***0*.*002***
Age, per year	1.01	0.98–1.04	0.62	―	―	―
Age >60 years	0.79	0.27–2.33	0.66	―	―	―
Performance status, per unit	1.81	1.27–2.57	***0*.*001***	―	―	―
Performance status ≥2	6.11	2.39–15.59	***<0*.*001***	―	―	―
Stage III/IV	2.54	0.94–6.91	0.067	―	―	―
Lactate dehydrogenase, per 100 IU/L	1.13	1.06–1.21	***<0*.*001***	―	―	―
Abnormal lactate dehydrogenase	2.44	0.57–10.54	0.23	―	―	―
Extranodal sites, per site	1.54	0.99–2.41	0.057	―	―	―
≥2 extranodal sites	3.47	1.13–10.62	***0*.*030***	―	―	―

DLBCL = diffuse large B-cell lymphoma.

^a^Adjusted for all variables for which hazard ratios are shown.

There is no national death registration in Malawi. Although patients are encouraged to return promptly for interim illnesses, deaths often occur at home or in facilities with limited diagnostic resources. Central adjudication of 35 deaths was undertaken to attribute cause (S1 Table). Twenty-three were attributed to lymphoma (12 HIV+, 11 HIV-), and 12 to CHOP treatment (9 HIV+, 3 HIV-). As shown, most treatment-related deaths occurred in patients with very adverse NHL characteristics.

## Discussion

This is the first prospective description of adults with aggressive NHL receiving frontline CHOP in SSA.

For HIV-infected NHL patients in Lilongwe, 50% OS at 12 months, and 59% among patients initiating CHOP, are among the best survival estimates reported at this time point from SSA [7, 14, 15]. This is a testament to the Malawi HIV program, which provides ART to 67% of HIV-infected individuals who require treatment [17]. Although 84% of our patients were on ART, median ART duration at lymphoma diagnosis was only 9.9 months with median CD4 count 121 cells/μL, highlighting a need for continued scale-up and earlier ART application. ART coverage, immune status, and HIV control in our cohort are comparable to similar routine-care populations in the US [20]. Although HIV-infected NHL patients with excellent outcomes similar to HIV-negative individuals have been repeatedly described in clinical trials from resource-rich settings (8–13), our prior work in a multicenter routine-care cohort in the US demonstrated a CD4 count of 123 cells/μL and HIV RNA suppression in only 28% at DLBCL diagnosis, among 201 patients with HIV-associated DLBCL receiving care in the ART era between 1996 and 2010, with 56% two-year overall survival (20). These characteristics are fairly similar to our Malawi cohort.

Despite increased treatment-related toxicity and deaths in the Malawi HIV-infected NHL population, our experience attests to the potential safety, effectiveness, and feasibility of CHOP in the current era, when accompanied by standardized monitoring, dose adjustment, supportive care, and ART, as also suggested by other studies [14, 15]. Most deaths among HIV-positive patients were from NHL rather than treatment. Treatment-related mortality occurred primarily in patients with very adverse NHL characteristics, and might be reduced with supportive care refinements. In particular, hematopoietic growth factors, which exist as generic biosimilars although even these are still too expensive for the Malawi public sector, could substantially mitigate toxicity since neutropenia was by far the most frequent adverse event. To our knowledge, the only alternative regimen to CHOP for HIV-associated NHL in SSA with published data is the dose-modified oral chemotherapy regimen (lomustine, etoposide, cyclophosphamide, procarbazine). In 49 patients with HIV-associated NHL in Kenya and Uganda before ART was widely available, this treatment resulted in median survival of 12.3 months and 33% five-year overall survival. Of note, the trial had major exclusions with respect to performance status, anticipated life expectancy, blood counts, and liver/kidney function, resulting in only 33% of screened HIV-associated NHL patients being enrolled, making it difficult to draw direct comparisons with our unselected patient population in Lilongwe.

HIV-negative lymphoma patients in Malawi are very ill with advanced, bulky disease and poor outcomes. More than three-quarters of deaths were attributed to NHL rather than treatment in the HIV-uninfected group. Massive HIV-related investments have occurred in SSA in recent years, which has created a strong network of HIV clinics throughout Malawi. No such primary care network exists for HIV-negative individuals, who consequently suffer from delayed referral and diagnosis. Delays were multifactorial and typically occurred before presentation to our center, as we made concerted efforts to expedite diagnostic work-ups and treatment initiation once patients entered our care. Absent radiotherapy in Malawi also contributes to poor outcomes, particularly for HIV-negative patients, who tend to present with localized, bulky disease, as radiotherapy improves outcomes for bulky NHL compared with chemotherapy alone [21, 22]. Occurrence of particularly aggressive lymphoma subtypes including T-cell NHL among HIV-uninfected patients also resulted in poor survival. Equivalent outcomes for HIV-positive NHL patients in Malawi are consistent with Uganda data suggesting HIV was not associated with worse survival for NHL [23].

Study strengths include prospective, longitudinal follow-up of NHL cases confirmed using real-time consensus telepathology, supported by IHC and multiple US and Malawi pathologists. This is notable given that diagnostic pathology was unavailable in Lilongwe before July 2011, and only 18% of cases in the Malawi national cancer registry were pathologically confirmed in the most recent report [24]. Subsequent secondary review was also performed by US hematopathologists. Patients underwent detailed and systematic clinical characterization, and those with HIV received concurrent ART in a mature national program. Patients were actively followed, and no enrolled patient has been lost to date with a median follow-up of 14.9 months among patients still alive. Median follow-up in studies from the region ranges from 3.5 to 8.2 months [7, 14, 15], and survival can be significantly overestimated in SSA when patients are lost to follow-up [25, 26]. We also made efforts to standardize chemotherapy, including strategies aimed at frail patients to minimize induction deaths. Our study lacked major exclusions and achieved 95% enrollment among NHL patients presenting for care, of whom 86% initiated cytotoxic therapy. Other NHL studies from SSA have reported 26–67% of patients being excluded or untreated [7, 14, 15].

Limitations include referral bias intrinsic to the Malawi health system, given centralization of cancer services in Lilongwe and Blantyre, the two largest cities. Many NHL patients may die before presenting to a national teaching hospital. Similarly, patients with asymptomatic lymphadenopathy may not travel to central hospitals, leading to underrepresentation of less aggressive disease. Another limitation is absent death certification in Malawi, leading us to attribute causation through centralized review.

Our results suggest a global strategy to improve outcomes for NHL patients is possible. HIV investments should be leveraged for populations with and without HIV. Community and health care worker education can facilitate earlier referral and diagnosis. Supportive care should be standardized and refined, informed by better identification of complications during treatment, with resource-appropriate incorporation of hematopoietic growth factors. Protocol-guided chemotherapy with defined strategies for monitoring and dose adjustment should be adopted. Ensuring continuous chemotherapy supply and incorporating newer agents to provide better first-line and salvage treatment options is also important. We believe a dedicated study of rituximab in SSA is an urgent priority to demonstrate safety and efficacy in severely resource-constrained settings like Malawi. This is important in light of rituximab’s impending patent expiration, successful use of an existing biosimilar, and increasing experience with subcutaneous administration which enhances feasibility for SSA [27, 28]. Non-radiotherapy strategies for localized bulky NHL also deserve investigation given radiotherapy scarcity in SSA [29]. Finally, biologic insights can guide treatment, and we have begun molecular profiling and virologic studies of Malawi NHL specimens to be reported subsequently.

In conclusion, CHOP can be safe, effective, and feasible for aggressive NHL patients with and without HIV in resource-limited settings in SSA. Among HIV-positive patients, ART use, CD4 count, and HIV RNA at lymphoma diagnosis are comparable to contemporary HIV-associated NHL cohorts in the US, as are outcomes for patients treated with CHOP. Multifaceted approaches are needed to improve survival, and these should target both HIV-infected and HIV-uninfected individuals.

## Supporting Information

S1 Table Central adjudication of deaths among aggressive non-Hodgkin lymphoma patients in Lilongwe, Malawi. (DOCX)
