# Supplementary material for: CHOP Chemotherapy for Aggressive Non-Hodgkin Lymphoma with and without HIV in the Antiretroviral Therapy Era in Malawi
Source: PLoS One. 2016 Mar 2;11(3):e0150445. doi: 10.1371/journal.pone.0150445 (PMC4775030; doi:10.1371/journal.pone.0150445)
Supplement: S1 Table — (DOCX) [file pone.0150445.s001.docx]

**Supplemental Table. Central adjudication of deaths among aggressive non-Hodgkin lymphoma patients in Lilongwe, Malawi.**

| Patient | CHOP | Disease | Comments |
| --- | --- | --- | --- |
|  |  |  | *Deaths before CHOP initiation* |
| 1 |  | X | HIV- stage IV anaplastic large cell lymphoma with bulky abdominal disease and malignant ascites. Received prephase prednisone but did not return for chemotherapy despite repeated attempts to contact. |
| 2 |  | X | HIV+ stage IV Burkitt lymphoma, performance status 3. Died before pathologic confirmation and chemotherapy initiation. |
| 3 |  | X | HIV+ stage IV DLCBL. Died of acute airway obstruction despite high-dose steroids before chemotherapy initiation. |
| 4 |  | X | HIV+ bulky DLBCL, CD4 <50, performance status 3. Presented with serum creatinine 15.8 mg/dL. Received prephase prednisone and medical optimization but died before chemotherapy initiation. |
| 5 |  | X | HIV+ stage IV Burkitt lymphoma, CD4 <50, performance status 3. Presented with renal failure, serum creatinine 2.4 mg/dL. Died before chemotherapy initiation. |
| 6 |  | X | HIV+ bulky cervical DLBCL. Developed acute airway obstruction requiring emergency tracheostomy and high-dose steroids. Died before chemotherapy initiation. |
| 7 |  | X | HIV- stage IV DLBCL. Died before pathologic confirmation and chemotherapy initiation. |
| 8 |  | X | HIV+ stage IV high-grade B-cell lymphoma, CD4 <50, performance status 4. Received prephase prednisone, died before chemotherapy initiation. |
|  |  |  | *Deaths after CHOP initiation* |
| 9 | X |  | HIV+ stage IV DLBCL with marrow involvement, CD4 <100, died 2 weeks after CHOP1. |
| 10 |  | X | HIV- stage III DLBCL completed CHOPx8 without grade 3/4 toxicity. Achieved partial response but progressed 3 months after CHOP completion. Received 2 salvage chemotherapy cycles with continued progression. |
| 11 |  | X | HIV+ stage IV DLBCL, CD4 >500, with marrow involvement. Achieved partial response but progressed 3 months after CHOP completion. Received 2 salvage chemotherapy cycles with continued progression. |
| 12 |  | X | HIV- with abdominal DLBCL progressed through CHOPx5. Died 1.5 months after last chemotherapy with no grade 3/4 toxicity encountered during treatment. |
| 13 | X |  | HIV+ stage IV DLBCL, CD4 >200, with marrow and leptomeningeal involvement. Achieved partial response, but died 3 weeks after CHOP5 with no grade 3/4 toxicity previously encountered. |
| 14 |  | X | HIV- with abdominal extranodal NK/T-cell lymphoma progressed through CHOPx4 eventually causing bowel obstruction. Died 1.5 months after last chemotherapy with no grade 3/4 toxicity encountered during treatment. |
| 15 | X |  | HIV- stage III bulky DLBCL, achieved complete response after CHOP1. Completed CHOPx3 but developed severe nausea/vomiting, dehydration, hypovolemic hypernatremia requiring hospitalization after cycles 2-3. |
| 16 |  | X | HIV- extranodal NK/T-cell lymphoma with orbital and leptomeningeal involvement refractory to CHOPx3. Died 1 month after last chemotherapy with seizures reported prior to death. |
| 17 |  | X | HIV- stage II bulky high-grade B-cell NHL refractory to CHOPx5. Received 2 salvage chemotherapy cycles with continued progression. |
| 18 | X |  | HIV+ DLBCL with palatal/central nervous system involvement, CD4 <100, died 1.5 weeks after CHOP1. |
| 19 |  | X | HIV- stage IV DLBCL completed CHOPx6 with complete response. Pathologically confirmed relapse 3 months after CHOP completion. Received 1 salvage chemotherapy cycle without response. |
| 20 |  | X | HIV+ plasmablastic lymphoma of oral cavity, CD4 <200. Completed CHOPx6 with complete response. Pathologically confirmed relapse 4 months after CHOP completion. Received 5 salvage chemotherapy cycles with partial response. |
| 21 |  | X | HIV+ bulky DLBCL, CD4 <100. Received CHOPx5 with partial response and prolonged neutropenia prohibiting CHOP6. Developed disseminated progression 3 months after CHOP5. Received 2 salvage chemotherapy cycles with partial response. |
| 22 |  | X | HIV+ stage IV DLBCL, CD4 >500. Received CHOPx8 with partial response. Progressive disease 5 months after CHOP8. Received 5 salvage chemotherapy cycles with continued progression. |
| 23 |  | X | HIV- palatal extranodal NK/T-cell lymphoma with sinus invasion died 2.5 months after CHOP1. Did not return for subsequent visits despite repeated attempts to contact. |
| 24 |  | X | HIV- stage IV DLBCL presented with spinal cord compression and nonambulatory status for 1 month. Died 2 months after CHOP1, did not return for subsequent visits despite repeated attempts to contact. |
| 25 | X |  | HIV+ stage IV aggressive B-cell lymphoma with marrow involvement, CD4 <50. Treatment complicated by severe immune reconstitution inflammatory syndrome after recent antiretroviral therapy initiation. Died 2 weeks after CHOP1. |
| 26 | X |  | HIV+ stage IV DLBCL with marrow involvement, CD4 >350. Died <1 week after dose-reduced CHOP1. |
| 27 | X |  | HIV+ stage IV DLBCL with marrow involvement, CD4 <100, achieved CR after CHOPx4 and could not receive additional cycles due to prolonged neutropenia. Died 9 months after last chemotherapy, seen 1 month before death at which time still in clinical remission. |
| 28 | X |  | HIV+ stage IV DLBCL, CD4 <100, performance status 3. Presented with hemoglobin 7 g/dL and creatinine 3 mg/dL. Partial response and improved performance status after dose-reduced CHOP1. Grade 4 anemia after full-dose CHOP2 leading to death. |
| 29 |  | X | HIV- plasmablastic lymphoma progressed through CHOPx4. Died 3.5 months after last chemotherapy. |
| 30 | X |  | HIV- stage II bulky DLBCL achieved complete remission after CHOPx8. Died 1.5 weeks after last chemotherapy cycle. |
| 31 | X |  | HIV+ stage IV aggressive B-cell lymphoma, performance status 4, CD4 >500. Presented with hemoglobin 7 g/dL and creatinine 6 mg/dL. Required tracheostomy and high-dose steroids for threatened airway. Died <1 week after dose-reduced CHOP1. |
| 32 |  | X | HIV+ stage IV DLBCL, CD4 >200, with refractory systemic disease to CHOPx6 and cytologically confirmed leptomeningeal progression. Died 3 weeks after CNS progression confirmed. |
| 33 |  | X | HIV+ stage III DLBCL, CD4 <200, with progressive abdominal disease after CHOP4 resulting in grade 4 obstructive renal failure. Died 2 months after last chemotherapy dose. |
| 34 | X |  | HIV+ stage III aggressive B-cell lymphoma, CD4 <50, achieved complete remission after CHOPx6. Died 1 month after last chemotherapy with family reporting anemia and no relapsed mass. |
| 35 | X |  | HIV- bulky stage III DLBCL. Died 2 weeks after dose-reduced CHOP1. |
| Total | 12 | 23 |  |

DLBCL=diffuse large B-cell lymphoma.
